# A novel grid-assisted pie-crusting technique for achieving soft tissue balance in total knee arthroplasty

**DOI:** 10.3389/fsurg.2025.1566642

**Published:** 2025-03-18

**Authors:** Qisheng Cheng, Yang Wang, Yi Liu, Jie Mu, Zhenyan Wang, Xu Lin, Guanchen Yin, Shuqiang Li

**Affiliations:** ^1^ Department of Orthopedic Center, The First Hospital of Jilin University, Changchun, China; ^2^ The First Operation Room, The First Hospital of Jilin University, Changchun, China

**Keywords:** arthroplasty, pie-crusting, medial collateral ligament, TKA, soft tissue balancing

## Abstract

**Background:**

To evaluate the effectiveness of a novel grid-based pie-crusting technique for soft tissue release at different locations of the medial collateral ligament (MCL) during total knee arthroplasty (TKA).

**Methods:**

Twelve fresh-frozen cadaveric knee joints were dissected. A novel grid was designed to cover the entire surface of the MCL. The specimens were divided into two groups: Group A, where only the central portion of the ligament underwent pie-crusting release, and Group B, where selective release targeted the femoral and tibial attachment points of the MCL. Mechanical testing was conducted via a Shimadzu AG-X precision instrument. Each group underwent twelve punctures, and data were collected to calculate deformation and stiffness metrics. The mean elongation and stiffness values were analyzed, and regression analysis was performed to evaluate correlations between the number of punctures and changes in elongation and stiffness.

**Results:**

No significant differences in initial stiffness were observed between the two groups (*P* = 0.42). Following 12 punctures, the stiffness decreased by 6.47 ± 4.06 N/mm in Group A and 1.08 ± 1.32 N/mm in Group B (*P* = 0.006). Despite this disparity in stiffness reduction, no significant differences in MCL elongation were observed between the groups. Group A demonstrated an elongation of 0.171 ± 0.180 mm, whereas Group B exhibited an elongation of 0.164 ± 0.123 mm (*P* = 0.47). A linear relationship was identified between stiffness reduction and the number of punctures (*R*^2^ = 0.61 ± 0.29), as well as between ligament elongation and the number of punctures (*R*^2^ = 0.89 ± 0.09).

**Conclusion:**

The grid-assisted pie-crusting technique, which uniformly covers the MCL, enables precise and controlled soft tissue release. This approach provides valuable insights for clinicians performing MCL release during TKA, facilitating improved soft tissue balance and potentially enhancing surgical outcomes.

## Introduction

Osteoarthritis (OA) is a prevalent degenerative joint disease worldwide. It is characterized by cartilage degradation, subchondral bone remodeling, and synovitis, primarily affecting the hip, knee, and hand joints, often resulting in pain and restricted mobility (1, 2). Among these conditions, knee osteoarthritis (KOA) is the leading cause of disability associated with OA globally (3). To address this condition, total knee arthroplasty (TKA) has become an increasingly common surgical intervention. In the United States alone, projections estimate that over 1 million TKA procedures will be performed annually within the next decade (4). The success of TKA depends significantly on achieving proper soft tissue balance during surgery. Alarmingly, some studies predict that the revision rate for TKA could increase to as high as 78%–182% by 2030 (5). Notably, complications arising from knee imbalance and instability constitute a substantial portion of revision cases (6).

Achieving appropriate soft tissue balance is critical for reducing the risk of early surgical failure, enhancing postoperative mobility and function, and improving functional scores within six months postoperatively (4, 7). Conversely, excessive soft tissue release may result in joint stiffness, prosthetic instability, persistent pain, and effusion (8). Intraoperatively, optimal knee balance is defined as an internal‒external load difference of less than 15 pounds, ensuring symmetric tension between the medial and lateral compartments and achieving balance in the flexion‒extension gap (9).

In TKA, common knee deformities include valgus and varus alignment, with varus deformity being the most prevalent, accounting for 68%–80% of cases (9–11). Despite thorough osteophyte removal and initial soft tissue release during conventional osteotomies, medial tension imbalances often persist, necessitating further medial soft tissue release to achieve precise alignment and balance. This process frequently involves stepwise release of the superficial medial collateral ligament (sMCL), deep medial collateral ligament (dMCL), semimembranosus tendon, posterior medial capsule, and pes anserinus tendon (12, 13). Ranawat et al. proposed the use of a scalpel to incrementally puncture the taut sites of the MCL until the desired correction is achieved in full knee extension (3). However, this method demands a high level of technical expertise to achieve accurate gap balancing while avoiding the overrelease of soft tissues.

Over the past fifteen years, the pie-crusting technique has been widely adopted for lateral soft tissue release in TKA (14). This method involves creating a series of horizontal incisions or punctures in the lateral collateral ligament (LCL) to gradually lengthen and adjust ligament tension. Pie crushing has since gained popularity for its efficacy in addressing soft tissue imbalance (15). Recently, its application has been extended to the medial collateral ligament (MCL), offering the advantage of preserving the structural integrity of the ligament (16). Previous studies have validated the safety and efficacy of pie-crusting via 16-gauge and 18-gauge spinal needles (15). Notably, Kwak et al. reported that 18-gauge spinal needles are safer than scalpels for this procedure (17), and that the optimal spacing between punctures should range from 3 to 5 mm (18, 19). Nevertheless, there remains a lack of definitive research regarding the precise puncture sites used during pie-crusting.

To address this gap, researchers developed a grid measuring 3 mm × 3 mm, designed to cover the entire surface of the MCL uniformly. Using an 18-gauge spinal needle, this grid facilitates precise and evenly distributed punctures. The present study aims to evaluate the safety and effectiveness of this novel grid-based pie-crusting technique at different sites of the medial collateral ligament, providing a more systematic and controlled approach to soft tissue release.

## Materials and methods

Knee joints from twelve fresh frozen cadavers were obtained from 6 donors, with an average age of 68 years (range, 61–75). All the samples were wrapped in gauze, stored in a −80 °C freezer, and then thawed for 24 h at 20 °C before testing. The knees from twelve cadaver samples were dissected, limbs showing history of knee surgery or clinical deformity were excluded, and median parapatellar arthrotomy along with tibial and femoral bone cuts were performed by experienced surgeons from our institution. The femur and tibia tissues were separated, and the MCL was carefully dissected to ensure that the femur and tibia were connected by independent MCL ligaments. The left and right knee joints from the same cadaver were assigned to different groups. We divided the knee joint samples into two groups: Group A, which released only the central portion of the ligament during pie-crusting, and Group B, which released only the femoral and tibial beginning points of the MCL.

**Figure 1:**
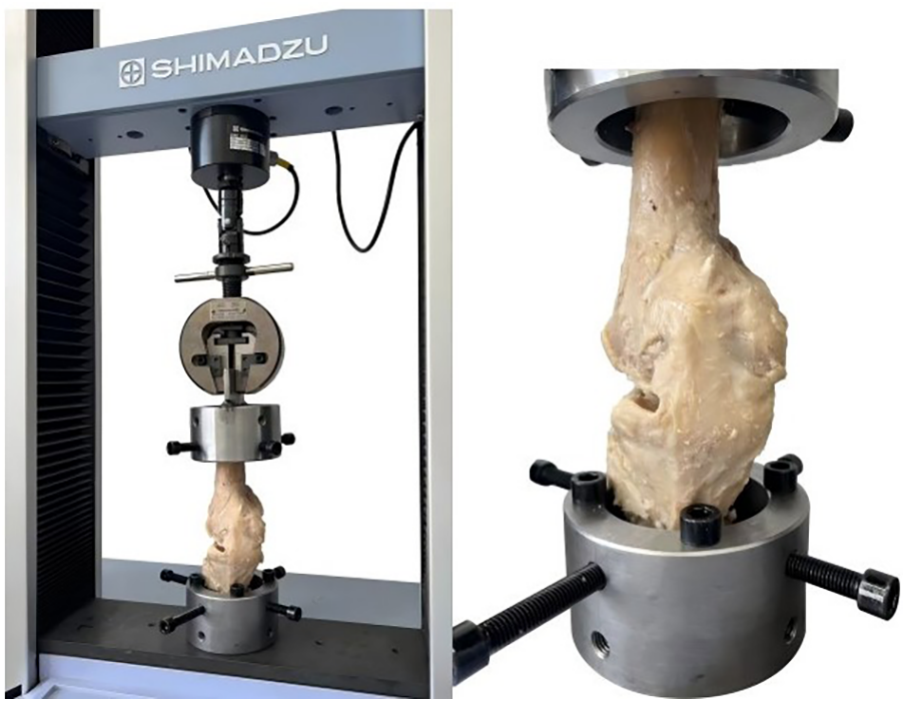
The custom-made fixtures secured the specimens in place and adequately aligned the knees for vertical tension.

In the experimental phase, tibial and femoral distal cuts similar to those performed in TKA were made. From thawing to mechanical testing, all the knee joint samples were kept moist with saline. As shown in Figure 1, the femoral and tibial distal ends were fixed via a customized fixture attached to an Instron AG-X precision universal testing machine. The knee joint was aligned to achieve vertical tension.

**Figure 2:**
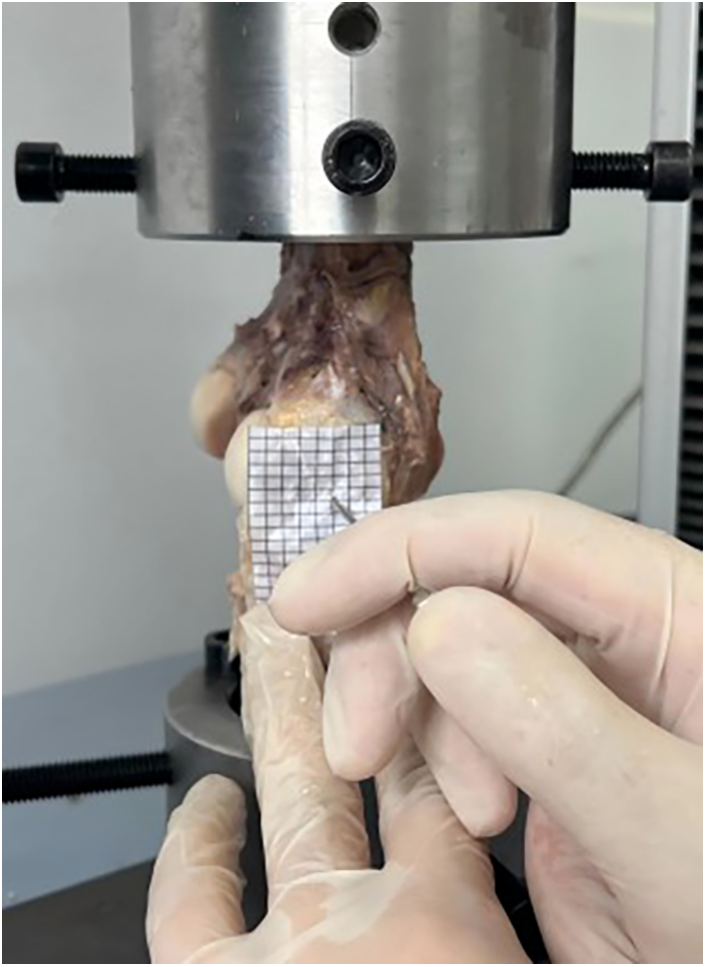
For accurate perforation via an 18-gauge needle, a grid covers the surface of the MCL.

Initially, a preloading force of 25–50 N was applied to the samples five times. On the basis of previous studies, a force of 80 N was chosen for this experiment (20). Moreover, a custom-made grid was used to cover the entire surface of the ligament. The grid served as a reference during the entire puncture process. The puncture needle was inserted perpendicularly to the MCL fibers from the outside to the inside. A total of twelve punctures were performed, with 4 holes in each row and 3 holes in each column. The distance between each hole was 3 mm, which also corresponded to the distance between the points on the grid (Figure 2).

After each puncture, software was used to collect data on ligament elongation, and the resulting deformation and stiffness were calculated. All the data were compared to the initial length and stiffness of the MCL. The average elongation and stiffness of the MCL were calculated, and regression analysis was performed to determine the correlation between the number of punctures and the elongation rate and stiffness of the MCL. These comparisons were performed via an independent samples *t* test. The linear relationship between the number of punctures and the elongation rate and stiffness of each sample was tested, and the R^2^ value was calculated.

## Results

**Figure 3:**
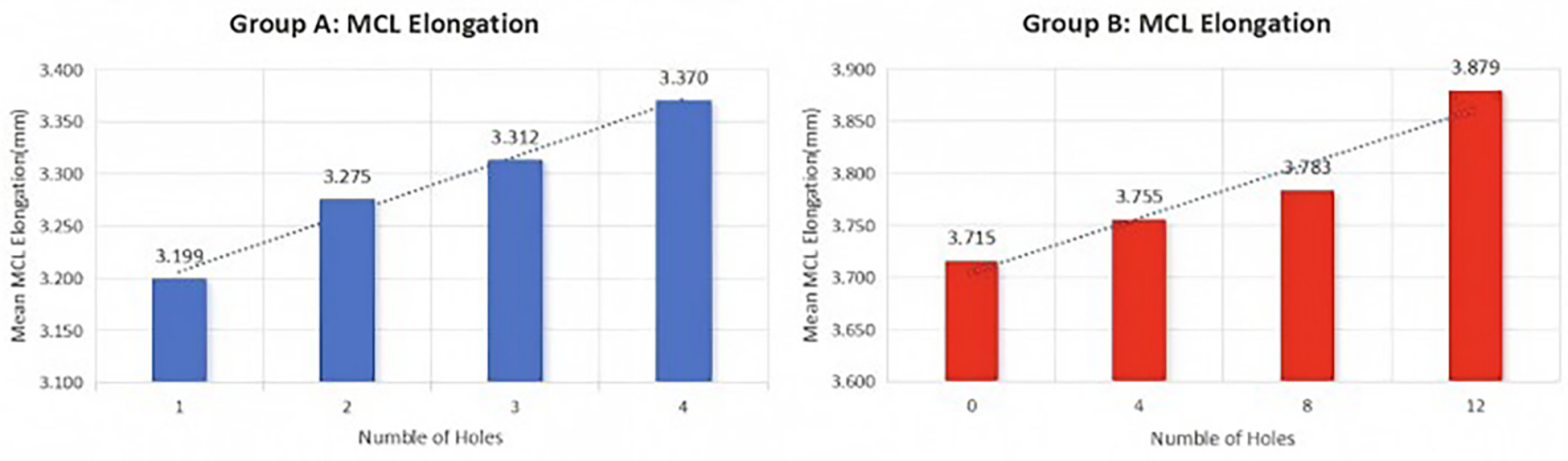
Change in MCL elongation with increasing number of perforations during pie-crusting.

There were no statistically significant differences in the initial stiffness of the ligaments between the two groups (Group A: 35.71 ± 2.87 N/mm; Group B: 36.17 ± 4.96 N/mm, *P* = 0.42). After the first puncture, the stiffness of the ligaments was as follows: Group A: 30.16 ± 8.93 N/mm; Group B: 35.33 ± 2.83 N/mm (*P* = 0.20), indicating no difference in stiffness after the first puncture. After the second puncture, there were also no differences between the two groups: Group A: 29.86 ± 9.16 N/mm; Group B: 34.47 ± 2.77 N/mm (*P* = 0.26). After the third puncture, Group A had 29.70 ± 8.61 N/mm, and Group B had 34.63 ± 2.75 N/mm (*P* = 0.21), with no significant difference. However, after 12 punctures, the change in stiffness relative to the initial stiffness significantly differed: Group A: 6.47 ± 4.06 N/mm; Group B: 1.08 ± 1.32 N/mm (*P* = 0.006). Table 1 shows the postpie-crusting data for both groups. Furthermore, the average stiffness of the ligaments in both groups decreased with increasing number of pie-crusting punctures (Figure 3).

**Table 1:** MCL stiffness with an increasing number of perforations during pie-crusting.

Group A: Stiffness (N/mm)					Group B: Stiffness (N/mm)				
Number of Holes	0	4	8	12	Number of Holes	0	4	8	12
KNEE 1	32.85	25.78	25.36	25.60	KNEE 7	37.54	36.43	33.48	34.65
KNEE 2	29.59	18.95	17.55	18.65	KNEE 8	36.38	35.41	33.76	33.81
KNEE 3	37.72	28.87	29.36	27.87	KNEE 9	32.35	32.35	32.24	32.58
KNEE 4	40.34	40.03	39.50	38.80	KNEE 10	31.88	31.57	31.58	31.10
KNEE 5	33.76	25.58	25.78	26.02	KNEE 11	37.64	37.88	37.64	37.49
KNEE 6	42.74	41.74	41.60	41.24	KNEE 12	38.50	38.39	38.15	38.15

**Figure 4:**
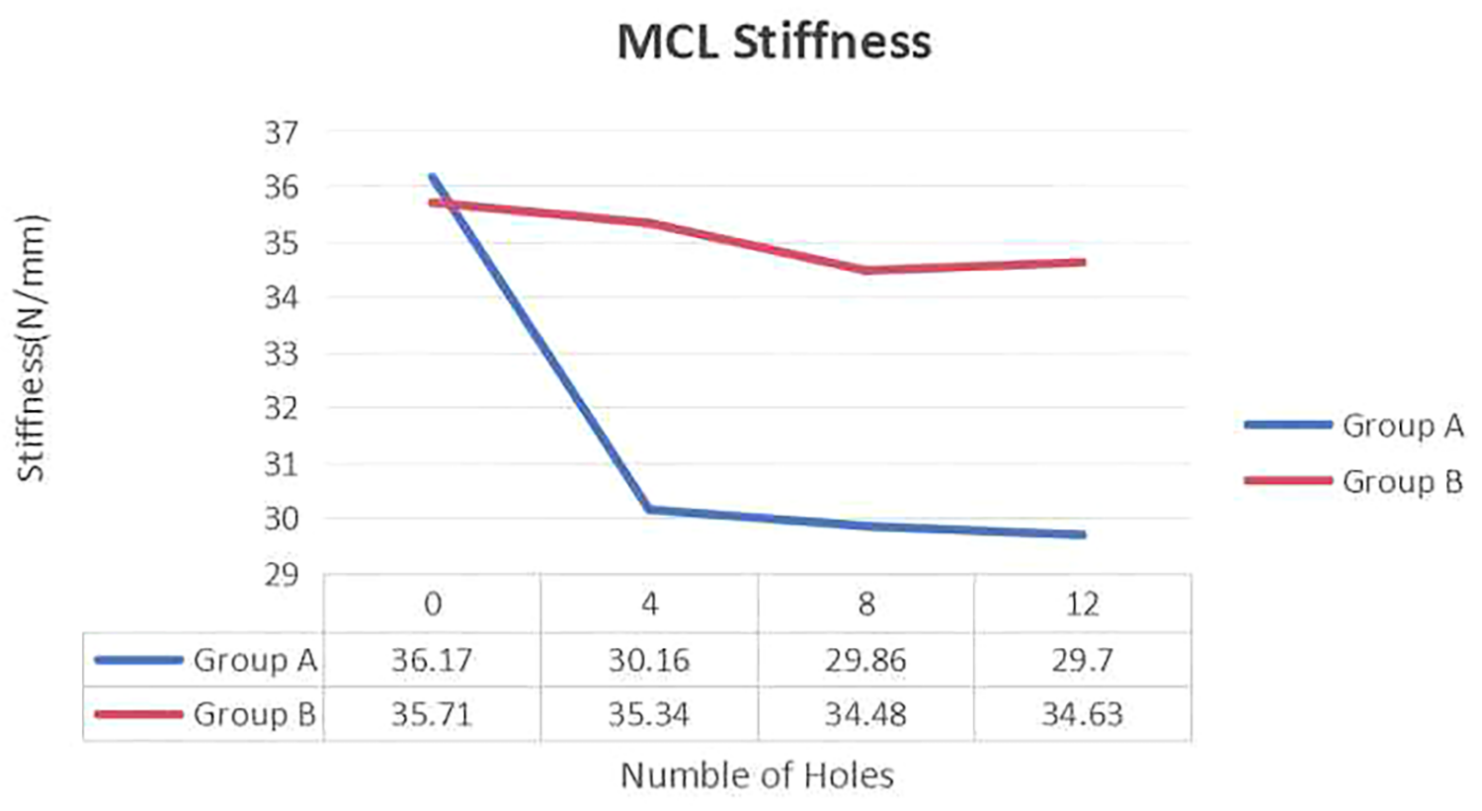
Change in MCL stiffness with increasing number of perforations during pie-crusting.

After the first puncture, the ligament elongation was as follows: Group A: 0.040 ± 0.018 mm; Group B: 0.076 ± 0.114 mm (*P* = 0.23). After the second puncture, Group A: 0.028 ± 0.021 mm; Group B: 0.037 ± 0.013 mm (*P* = 0.38). After the third puncture, Group A: 0.096 ± 0.102 mm; Group B: 0.058 ± 0.068 mm (*P* = 0.46). There were no significant differences in elongation rates among the groups after each puncture. After all the punctures, the average elongation for Group A was 0.171 ± 0.180 mm, and for Group B, it was 0.164 ± 0.123 mm (*P* = 0.47). Table 2 shows the elongation data after pie-crusting for both groups. The average elongation rates of the MCL in Group A and Group B were linearly related to the number of punctures (Figure 4).

**Table 2:** MCL elongation with an increasing number of perforations during pie-crusting.

Group A: MCL Elongation (mm)					Group B: MCL Elongation (mm)				
Number of Holes	0	4	8	12	Number of Holes	0	4	8	12
KNEE 1	3.682	3.975	4.022	4.213	KNEE 7	5.399	5.439	5.492	5.563
KNEE 2	2.829	2.935	2.976	2.990	KNEE 8	5.921	5.984	6.032	6.323
KNEE 3	3.201	3.217	3.256	3.305	KNEE 9	2.845	2.868	2.880	2.998
KNEE 4	3.347	3.370	3.387	3.444	KNEE 10	2.321	2.337	2.359	2.401
KNEE 5	2.984	2.966	3.017	3.042	KNEE 11	3.632	3.674	3.709	3.751
KNEE 6	3.151	3.187	3.215	3.226	KNEE 12	2.172	2.227	2.226	2.239

These results suggest that puncturing at different regions of the MCL does not affect the rate of ligament elongation. However, differences were found in the stiffness reduction after puncturing at different locations. In addition, the number of punctures was linearly correlated with elongation (*R*^2^=  0.89 ± 0.09) and stiffness reduction (*R*^2^ = 0.61 ± 0.29).

## Discussion

Achieving optimal soft tissue balance in total knee arthroplasty (TKA) remains a complex and subjective challenge, yet it is crucial for preventing early failure of the procedure (21). Preoperative radiological assessment included standing hip-knee-ankle true antero-posterior and true lateral radiographs, and varus/valgus stress radiographs of the knee joint in full extension. Postoperative evaluation is usually performed by MRI of the knee joint. The adequacy of soft tissue release depends heavily on the surgeon's skill, experience, and tactile feedback. But the advent of sensor technology has enabled surgeons to utilise real-time data, thereby circumventing the potential for errors that may arise from subjective judgement (22). Traditional methods, such as those proposed by Insall, typically involve elevating the anterior fibers of the superficial medial collateral ligament (sMCL) while preserving the integrity of the pes anserinus tendon. However, this approach carries the risk of excessive release, potentially leading to medial collateral ligament (MCL) insufficiency and subsequent joint instability. Overrelease is a well-documented contributor to varus or valgus deformities, and it is responsible for approximately 22% of TKA revisions (23). Rosso et al. conducted a study that yielded findings which suggest that the implementation of a liner could serve as a preventative measure against aseptic postoperative loosening or surgical failure in cases where loosening occurs during surgery (24).

The pie-crusting technique, which was originally used for lateral soft tissue release, has gained widespread acceptance for medial soft tissue release because of its safety and efficacy, as demonstrated in both clinical and cadaveric studies (15). Dunbar's research confirmed that selective lateral structure release via pie-crusting effectively corrects foot valgus deformities (25), a principle that has also been successfully applied to the MCL (8). Consequently, pie-crusting is increasingly recommended by surgeons for controlled soft tissue release. Clarke et al. reported favorable outcomes in 24 cases where pie-crusting was employed during TKA, with no evidence of prosthetic instability or loosening after a 54-month follow-up and significant improvements in knee scores (6). Similarly, Bellemans et al. reported that in 35 patients treated with pie-crusting, only one patient required a thicker implant due to excessive release, while knee scores improved significantly from 41 to 93 (26). These findings align with the cautionary remarks of Kwak et al., who highlighted the potential for early excessive release during pie-crusting. No sign of unstable knee was observed during the 1–2 year follow-up of the pie-crusting technique performed on the MCL (15, 27).

In current study, researchers demonstrated that the grid-assisted pie-crusting technique, which uses an 18-gauge needle, effectively induced ligament elongation in a controlled and reproducible manner. A statistically significant linear relationship was observed between the number of punctures and ligament elongation after 12 punctures. Crucially, no significant differences in elongation were found between punctures performed at the center of the ligament and those at the femoral and tibial attachment points. This finding suggests that surgeons can target the most prominent regions of the ligament during surgery without the need to focus on a specific anatomical site. Although this technique is relatively simple to perform, it does require a certain amount of experience and skill on the part of the surgeon, particularly in selecting puncture sites and controlling the number of punctures. Surgeons new to the technique may require some training to become proficient. Furthermore, the absence of significant differences in elongation between groups after each puncture indicates that the location of the pie-crusting puncture does not have a substantial effect on ligament elongation.

A particularly noteworthy finding from our study was the linear relationship between the number of punctures and the degree of ligament elongation. This finding suggests that the desired degree of elongation can be accurately controlled by adjusting the number of punctures, making this approach predictable and reproducible for surgeons. However, it is important to recognize that individual variations in ligament stiffness and deformity severity could influence the effectiveness of this technique. It is also possible that the nature of the fresh cadaveric MCL in this study differed from that of the intraoperative patient, because of the low metabolic rate and well-developed anaerobic energy-generation capacity (28). Leading a low metabolic rate results in slow healing after injury (29). This constitutes one of the rationales for which excessive ligament release is eschewed in TKA surgery. Therefore, a preoperative assessment of the ligament's characteristics is essential for the successful implementation of this method.

Moreover, both experimental groups showed a progressive decrease in stiffness with increasing number of punctures; however, the structural integrity of the ligament was preserved. Notably, Group A, which underwent central puncturing, experienced a significantly greater reduction in stiffness than Group B did. This finding suggests that puncturing the center of the ligament may have resulted in a more pronounced impact on ligament stiffness. In contrast, puncturing near the femoral and tibial attachment sites seems to achieve elongation with less disruption to the ligament's structural integrity. These findings imply that targeting the peripheral regions of the ligament could help minimize the risk of postoperative instability. However, as our study focused solely on the MCL, further research is needed to assess whether similar results would be observed in clinical settings involving additional tissues around the knee joint.

Results also highlight the importance of customizing the pie-crusting technique to individual patient characteristics. Variations in ligament width and fiber composition may influence both elongation and stiffness outcomes. Previous studies have shown that narrower ligaments tend to elongate more and have lower failure loads than wider ligaments under the same number of punctures (30). This underscores the necessity of preoperative evaluation to optimize soft tissue release strategies for each patient. Clinicians are also required to fully inform patients about treatments, treatment alternatives and major risks prior to surgery (31).

Furthermore, advancements in robotic technology not only offer a useful tool for revising UKA to TKA, but the potential for more precise ligament release tailored to the individual's knee anatomy (32). Preoperative 3D scanning can aid in determining the optimal implant position and size (33), whereas robotic systems enable intraoperative, quantitative assessment of soft tissue balance, allowing for precise adjustments to restore optimal joint function (34). Early studies have shown promising results in the use of robotic systems for knee joint gap balancing, alignment restoration, and postoperative functional improvement (35–36). A recent study suggests that patients’ range of motion and subjective and objective scores improved significantly when advanced robotic systems were used to treat severe knee deformities (37). However, robotic platforms remain expensive and require extensive training, making them inaccessible for many surgeons. Consequently, for those unable to access such technology, more effective manual methods, such as grid-assisted pie-crusting, provide a viable and cost-effective alternative for achieving soft tissue balance (38).

Several limitations of this study should be acknowledged. First, the use of freshly frozen cadaveric samples introduces potential variability, as multiple freeze‒thaw cycles at −80 °C could alter tissue properties. Although only one freeze‒thaw cycle was used in this study, the impact on the samples is believed to be minimal, also impossible to study the healing of the ligaments (39, 40). Additionally, the sample size of 12 knee joints, which can be divided into two groups, is relatively small for clinical studies, although small sample sizes are common in biomechanical research. The pie-crusting technique in this study was applied to normal knee joints; thus, its effects on patients with varus or valgus deformities were not evaluated. Further research is needed to determine whether similar results would be obtained in clinical patients. Additionally, the study did not account for variations in age or sex, which may affect the relative stiffness of ligaments. Since the MCL was the only structure preserved in this study, the generalizability of the findings to patients undergoing total knee arthroplasty with a complete prosthetic knee structure is limited. And the implementation of the grid-assisted pie-crusting technique is not without its challenges. Surgeons must navigate the complexities of tissue, including tissue thickness, vascularity and surrounding structures. This can make precise grid placement and uniform puncturing more difficult compared to cadaveric studies. Finally, all the experiments were conducted with the knee in a fully extended position, and the effects of pie-crusting in a flexed knee position were not explored. Future studies should assess the impact of pie-crusting on soft tissue release during knee flexion, as this may further refine intraoperative soft tissue balancing techniques.

In conclusion, present study demonstrated that the grid-assisted pie-crusting technique for MCL release is both safe and effective. It offers a controlled method of gradually elongating the MCL without compromising the structural integrity of the ligament, thus minimizing the risk of postoperative instability associated with excessive release. The technique also provides flexibility, allowing surgeons to select the optimal puncture sites on the basis of the individual patient's anatomical features. While robotic systems offer advanced capabilities for soft tissue balancing, the grid-assisted pie-crusting technique remains a cost-effective and accessible alternative with significant clinical value. Further research is needed to explore its application in clinical patients, particularly those with knee deformities and during knee flexion, to better refine and optimize this promising technique.

## Data Availability

The original contributions presented in the study are included in the article/Supplementary Material, further inquiries can be directed to the corresponding author.
